# The Relationship Between Inflammation and Post-traumatic Stress Disorder

**DOI:** 10.3389/fpsyt.2021.707543

**Published:** 2021-08-11

**Authors:** Yajing Sun, Yuanyuan Qu, Jianwei Zhu

**Affiliations:** ^1^West China Biomedical Big Data Center, West China Hospital, Sichuan University, Chengdu, China; ^2^Med-X Center for Informatics, Sichuan University, Chengdu, China; ^3^Department of Orthopaedic Surgery, West China Hospital, Sichuan University, Chengdu, China

**Keywords:** traumatic stress, stress disorders, PTSD, stress-immune disorders, inflammation

## Abstract

**Background:** Stress disorders, such as post-traumatic stress disorder (PTSD), are attracting much attention. However, the relationship between traumatic stress and inflammation is rarely discussed.

**Subjects and Methods:** As studies have linked PTSD to altered susceptibility to various diseases, such a psychiatric condition may lead to long-term systematic changes in physiological functions. We searched PubMed with the keywords “traumatic stress,” “stress disorders,” “post-traumatic stress disorder,” and “inflammation.”

**Results:** Based on 65 previously published studies, we reviewed the long-term effects of PTSD, as well as traumatic events, on inflammatory function from both epidemiological and biological perspectives. Post-traumatic stress disorder is related to the immune response, including an increase in inflammatory factors and a reduction in anti-inflammatory factors. Additionally, it has been demonstrated that traumatic stress disorder and immune disease share a common genetic basis at the gene expression level.

**Conclusions:** Understanding this relationship is of great significance for optimizing treatment plans for patients with PTSD.

## Introduction

Traumatic stress includes post-traumatic stress disorder (PTSD), acute stress disorders, reactive attachment disorder, disinhibited social engagement, and adjustment disorders diagnosed based on the International Classification of Diseases (ICD) and/or the Diagnostic and Statistical Manual of Mental Disorder (DSM). It is characterized by obvious changes in the body's physiological functions and increases the medical burden in common worldwide (1–3).

Post-traumatic stress disorder is a mental disorder that can occur in people who have experienced or witnessed traumatic events or threatened with death, sexual violence, or serious injury (4). Acute stress disorder is a response to traumatic events and has symptoms similar to PTSD. It is hoped to predict the development of PTSD in patients with acute trauma so that early intervention can be initiated (5). They are estimated to be associated with subsequent major disease risk (6–8). Therefore, it is important to understand the mechanism of human physiological function changes or possible related indicators that may be caused by traumatic stress to improve treatment options for traumatic stress. Although the pathophysiology of traumatic stress is not yet fully understood, its correlation with immune disorders has been extensively studied (9–11).

**Figure 1:**
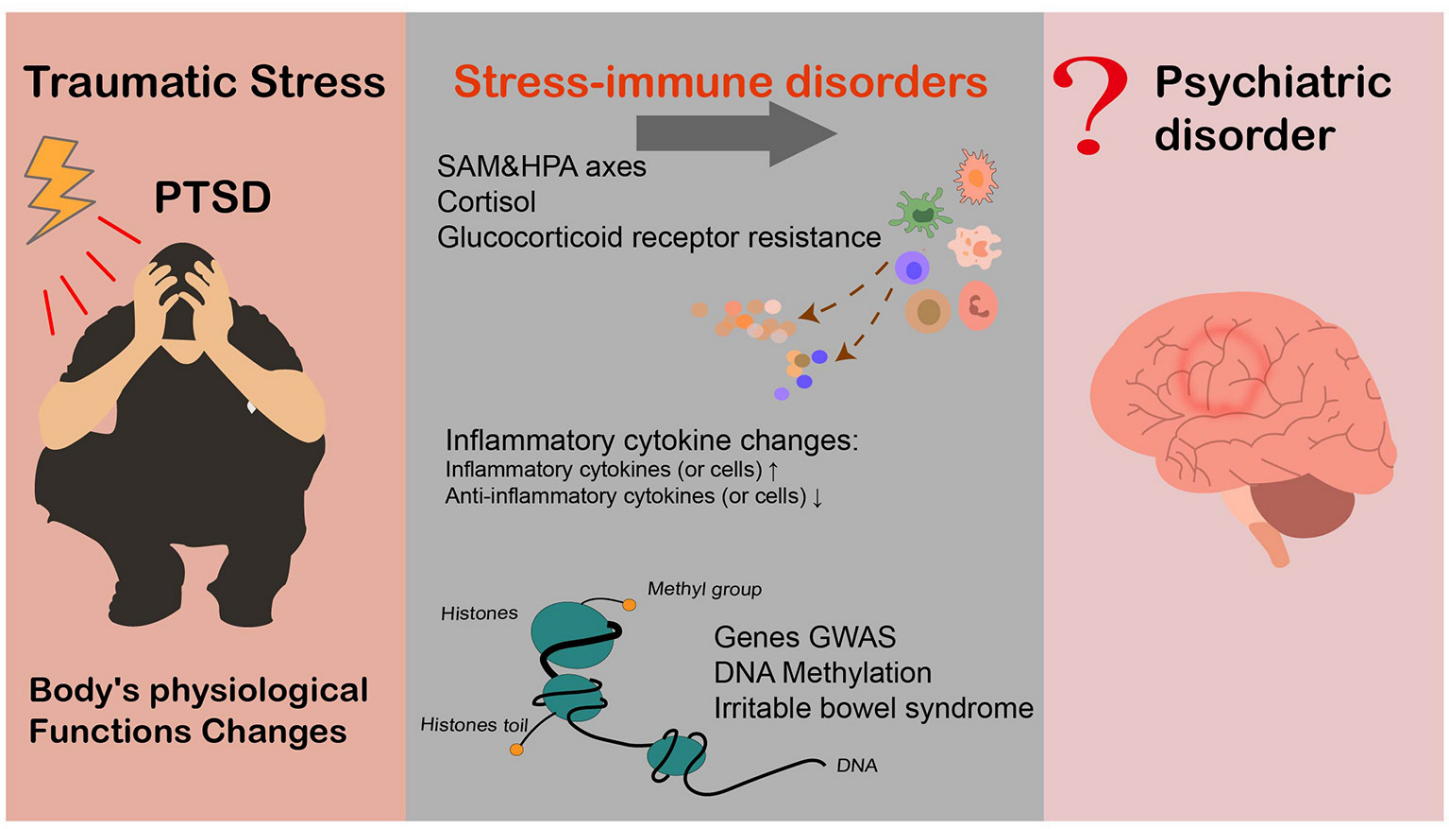
The relationship between inflammation and PTSD.

In addition, many findings point to a link between inflammation and depression. Stress-immune disorders include physiological mechanisms, immunological indicators of traumatic stress disorder, multiple mechanisms of PTSD, and immune dysfunction. In this review, we focus on the relationship between traumatic stress, mainly PTSD and inflammation (see Figure 1). We review the long-term effects of PTSD and traumatic events on inflammatory function (including peripheral inflammation and neuroinflammation) from an epidemiological and biological perspective. Traumatic stress disorder, mainly PTSD, is related to the immune response, including increases in inflammatory factors and decreases in anti-inflammatory factors. In addition, it has been demonstrated that PTSD and immune diseases have a common genetic basis at the gene expression level. Understanding this relationship is important for optimizing treatment plans for patients with traumatic stress disorder.

## Traumatic Stress Disorder may Cause Changes in the Body's Physiological Functions

As the scope of people's activities and communication expand, the probability of being exposed to traumatic events over the course of a lifetime may increase as well.

Accumulating studies have shown that exposure to traumatic events may have some adverse effect on body's physiological functions (12–14). For instance, symptoms including anxiety, being guilt for disable operation, mental numbness, and separation, and feelings of helplessness and loneliness have been observed among post-war soldiers (12). Veterans were found to have a higher risk of suffering from many major chronic diseases (i.e., circulatory, nervous, digestive, musculoskeletal, or respiratory diseases) after the war (13). Studies also shown that adolescents those who experienced stressful events may be worse school performance or have worse health conditions (14). The subsequent long-term physiological changes, especially inflammatory function disorders, may lead to a decline in overall health and an increase in diseases emergence and even death. Indeed, the adverse health consequences related to mental stress are especially obvious in those diagnosed with PTSD (9, 15–17). In contrast, some consider that traumatic events may engender positive effects, such as self-reported posttraumatic growth (18, 19).

According to Kessler and Breslau, more than 80% of individuals are exposed to at least one traumatic life event at some point in their lives (20, 21), including death of a loved one, diagnosis of a major illness, natural disaster, violence, assault, and discrimination, among others. Based on data from the United States, each person experiences an average of 4.8 significant traumatic events, while males (5.3) had more traumatic events than females (4.3) (21). Therefore, those who have experienced or are experiencing traumatic events compose a large group that cannot be ignored. Although many people can overcome the stress response caused by such traumatic events within a certain period, some individuals will develop mental stress disorder after traumatic events, resulting in impaired social functions and subsequent health problems (15, 16). Indeed, Song et al. conducted a series of nationwide cohort studies among Swedish individuals, suggesting that mental stress disorder caused by traumatic events (including acute stress disorder, PTSD, and adaptability obstacle) may be present during a very long period of follow-up (up to 30 years) (9). These authors also noted that long-lasting stress disorder might lead to an increase in the risk of autoimmune disease (9) and serious infectious diseases (22). In fact, the incidence rate of autoimmune diseases, such as lupus or rheumatoid arthritis, is higher among those who have been previously diagnosed with stress-related diseases (23). These findings suggest that mental stress disorder may cause long-term systematic changes in the body's physiological functions, such as disorders of the immune system, and thus alter susceptibility to various diseases.

## Research Significance of Changes in Physiological Function

Post-traumatic stress disorder, the most serious traumatic stress disorder, has been associated with subsequent major disease risk such as (24–27) and therefore has gained increasing attention worldwide in recent years. Accumulating evidence suggests a general health decline in those who experience PTSD, which suggests a substantial disease burden (1). In China, the diagnosis and treatment rates of PTSD are low. However, screenings conducted in specific groups, such as military personnel and earthquake survivors, have revealed that these high-risk populations had a PTSD incidence rate similar to that reported in Western countries. Furthermore, studies conducted by Song et al. have shown that (9, 22) in addition to PTSD, other mild forms of mental stress disorders, such as acute stress disorder and adaptive disorders, may induce physiological disorders and increase the risk of major diseases. Without specific reports on the prevalence of all stress disorders worldwide, studies have indicated 10 times more cases of any stress disorder than cases of PTSD (9, 22), highlighting the need to study physiological dysfunction, as well as its possible intervention, among this large and vulnerable group.

## Stress-Immune Disorders

### Physiological Mechanisms

Physiological mechanisms related to stress have been extensively studied. It is clear that the exposure to traumatic events can lead to the dysregulation of the sympathetic adrenaline-medulla (SAM) and hypothalamic-pituitary-adrenal (HPA) axes. Indeed, researchers have detected the persistent content variations as well as abnormal circadian secretion rhythms of cortisol, in individuals with a strong stress response. As a key endocrine regulator involved in immune and phlegm responses, cortisol may be an important factor in human immune dysfunction (11, 28). Additionally, a recent study showed (29) that the human body may develop glucocorticoid receptor resistance (GCR) under continuous pressure stimulation. This leads to chronic underlying inflammation in the body, which may subsequently promote the development of major somatic diseases, such as cardiovascular diseases.

### Immunological Indicators of PTSD

Biomarker studies have analyzed various immunological indicators among patients with traumatic stress disorder, especially those with PTSD. Although the results are somehow inconsistent, most studies have observed abnormalities in immune indicators among patients with PTSD. A summary of studies reporting cytokine testing are presented in Table 1. These results indicate an altered immune balance in PTSD patients. Specifically, both plasma levels of proinflammatory cytokines such as IFN-γ, IL-6, TNF-α, and IL-17 and blood levels of immune-stimulating Th1 and inflammatory Th17 cells increased, suggesting a proinflammatory status (29). Nevertheless, these studies generally had some deficiencies.

**Table 1:** Summary of literature reporting inflammatory cytokine changes in PTSD patients.

**Research findings**	**Sample source**	**Index**	**Reference**
Levels of inflammatory cytokines (or cells) ↑	Plasma	IL-2, IFN-γ, IL-6, TNF-α, IL-12, IL-17	(30–33)
	Serum	TNF-α, IL-1, IL-6, IFN-γ	(34)
		(TNF)-α, (IFN)-γ, and CRP	(35)
	Serum	TNF-α, IL-1β, and IL-6	(36)
	Serum	IL-1β	(37, 38)
	Serum	IL-4, IL-6, IL-10, and TNF-α	(32)
	blood sample	IL-6	(16, 39–41)
	serum	IL-6	(42, 43)
	Cerebrospinal fluid	IL-6	(44)
	Saliva	IL-2, IFN-γ, IL-6, IL-17, CCL2	(31, 45)
	Secretion of Blood cell	IL-1, IL-6, TNF-α	(46)
	Blood cell	Th1, Th17	(47)
Anti-inflammatory cytokines (or cells) ↓	Plasma	TGF-β, LI-4, IL-10	(30)
	Serum	IL-2, IL-8	(43)
	Saliva	IL-4, IL-10	(31)
	Blood cell	Treg	(46)

Most existing studies were performed in a small number of PTSD patients, rendering the findings inconclusive and difficult to generalize (30, 31, 33, 37, 39, 42, 44, 45).

The measurement of immune inflammatory factors is limited; most studies have only evaluated cytokine levels, with a few assessing the function of blood immune cells.

Large differences in background among populations and significant differences in geographical and socioeconomic backgrounds exist, which may affect quantitative results.

There are variations in the types and duration of traumatic events; for example, wars are relatively continuous, whereas car accidents, earthquakes, and volcanic eruptions are transient (48).

Most of the included PTSD cases were newly diagnosed, and thus, the long-term effects of traumatic stress disorder on inflammatory function remain to be determined.

Evidence is scarce regarding the effect of trauma and stressor-related disorders on neuroinflammation biomarkers. Post-traumatic stress disorder is a mental disorder caused by trauma. It is widely believed that its key mechanism is abnormal fear subsidence, which involves the biological dysfunction of the fear circuit area in the brain. Neuroinflammation after a single prolonged stress exposure may play a key role in the loss of impaired fear memories (49). However, the effect of PTSD on neuroinflammation biomarkers, including HMGB1 and Toll-like receptor 4 (50), IL-1β, and TNF-α (51), were mainly found in animal models. Related research on molecular indicators of stress-induced neuroinflammation was also found in a mouse model, which was a simulated feature of PTSD (52). The effects of trauma and stressor-related disorders on specific biomarkers of neuroinflammation deserve further investigation.

Evidence also has shown the effects of trauma and stressor-related disorders on neurodegeneration biomarkers. Studies have shown that PTSD was associated with the accumulation of amyloid beta (Aβ) and tau protein deposits, which may contribute to neurodegeneration in multiple forms of dementia (53). Exosomal neurofilament light may become a potential biomarker for remote symptoms after mild traumatic brain injury (54).

### Multiple Mechanisms of PTSD and Immune Dysfunction

Although PTSD is associated with immune dysfunction, the underlying mechanisms remain unclear. In 2010 (55), Sandro Galea and colleagues analyzed more than 14,000 genes using DNA from blood samples of 100 Detroit residents. Unusual DNA methylation levels were detected among people diagnosed with PTSD (*N* = 23), which were six to seven times higher than those without PTSD (*N* = 77). This was the first study exploring the effect of PTSD on the immune system from an epigenetic perspective. Another comprehensive epigenetic and blood cytological analysis showed that changes in the methylation levels of some gene (interferon gamma, IFNG, and IL-12b) promoters in peripheral blood monocytes might be responsible for the increase in inflammatory cytokines (such as IL-12) in PTSD patients (33). Altogether, these data suggest that traumatic events may disrupt the immune system by altering gene expression (E × G).

In addition, supportive evidence has been provided by recent studies suggesting an association of PTSD with irritable bowel syndrome (IBS) (56), a stress-related gastrointestinal and autoimmune disease. Early stress, especially PTSD, may lead to lasting changes in GCR genes, elevating proinflammatory cytokines such as IL-6 and IFN-A in IBS and promoting GCR resistance, thereby amplifying the initial epigenetic changes. Hypothalamic-pituitary-adrenal axis disorder and abnormal serotonin energy (5-HT) function resemble the mechanism of IBS (57, 58).

Finally, family-based and genetic analyses (such as genome-wide association studies, GWASs) of PTSD patients have confirmed the genetic basis of traumatic stress disorder. For instance, twin studies on the inheritance rate of PTSD showed that 24–72% of the PTSD risk may be determined by genetic factors (59, 60), and the inheritance rate of females was significantly higher than that of males (2–3 times) (61). To follow up on recent findings of psychiatric stress-autoimmune disorders, it is important to explore the role of genetic factors in the association between psychiatric stress disorder and autoimmune disorders. Previous GWASs have found important evidence of genetic pleiotropy between PTSD and rheumatoid arthritis and psoriasis (62). Moreover, in terms of gene expression level, enrichment correlation analyses have shown that schizophrenia (a mental disease with high genetic overlap with PTSD) and immune diseases exhibit clustering phenomena (63). Furthermore, functional analysis of transcriptome sequencing data by Bam et al. revealed abnormal expression of inflammation-related genes in PTSD patients, paralleling the dysfunction of the immune network (38). Overall, further study is warranted to investigate the shared genetic component of the stress-immune relationship (see Figure 1).

## Summary and Future Directions

Many recent studies have explored the negative impact of traumatic stress disorder on overall health after exposure to traumatic events, making traumatic stress disorder a new research focus and concern in the field of mental illness. To improve the level of diagnosis and treatment of traumatic stress disorder, especially PTSD, it is necessary to find a method to effectively diagnose and treat it. According to previous studies, PTSD is related to changes in the immune system, which may increase the level of inflammatory factors such as IFN-γ, IL-6, TNF-α, and IL-17 and a reduction in the level of anti-inflammatory factors (e.g., IL-4 and IL-10). Simultaneously, genetic studies involving DNA analysis of patient blood samples have found some unusual DNA methylation. Additionally, at the gene expression level, there is evidence that PTSD and immune disease share a common genetic basis, enabling the study of a genetic-based treatment approach of stress-immunity correlation.

With these findings (9, 22) indicating that early continuous drug therapy for PTSD, such as selective serotonin reuptake inhibitors (SSRIs), can reduce the risk of later related physical diseases, therapeutic interventions for PTSD may, to some extent, moderate immune dysfunction caused by PTSD (see Figure 1). The above mentioned evidence suggests that treating PTSD might improve stress-related immune damage. However, there is limited evidence for this view at present, and thus, experimental studies are urgently needed for and further verification. In addition, given that the treatment strategies of mental stress disorders are not well-established, further studies on various potential therapies are of great significance not only to alleviate the symptoms of traumatic stress disorder but also to prevent further health decline.

## Author Contributions

JZ and YS conceived and designed the study. YS wrote the paper by searching the literature. YQ reviewed and edited the manuscript. All authors read and approved the manuscript.

## Conflict of Interest

The authors declare that the research was conducted in the absence of any commercial or financial relationships that could be construed as a potential conflict of interest.

## Publisher's Note

All claims expressed in this article are solely those of the authors and do not necessarily represent those of their affiliated organizations, or those of the publisher, the editors and the reviewers. Any product that may be evaluated in this article, or claim that may be made by its manufacturer, is not guaranteed or endorsed by the publisher.
